# Association between first caesarean delivery and adverse outcomes in subsequent pregnancy: a retrospective cohort study

**DOI:** 10.1186/s12884-018-1895-x

**Published:** 2018-06-28

**Authors:** Hong-Tao Hu, Jing-Jing Xu, Jing Lin, Cheng Li, Yan-Ting Wu, Jian-Zhong Sheng, Xin-Mei Liu, He-Feng Huang

**Affiliations:** 1 Department of Obstetrics and Gynecology, International Peace Maternity and Child Health Hospital, School of Medicine, Shanghai Jiao Tong University, Shanghai, China; 2 Department of Reproductive Medicine, International Peace Maternity and Child Health Hospital, School of Medicine, Shanghai Jiao Tong University, Shanghai, China; 3 Department of Pathology and Pathophysiology, School of Medicine, Zhejiang University, Zhejiang, China; 4 Institute of Embryo-Fetal Original Adult Disease, Shanghai Key Laboratory for Reproductive Medicine, School of Medicine, Shanghai Jiao Tong University, Shanghai, China; 5 International Peace Maternity and Child Health Hospital, School of Medicine, Shanghai Jiao Tong University, 910 Hengshan Road, Shanghai, 200030 China

**Keywords:** Cohort, Pregnancy outcomes, Caesarean delivery, Subsequent pregnancy

## Abstract

**Background:**

Few studies have explored the association between a previous caesarean section (CS) and adverse perinatal outcomes in a subsequent pregnancy, especially in women who underwent a non-indicated CS in their first delivery. We designed this study to compare the perinatal outcomes of a subsequent pregnancy in women who underwent spontaneous vaginal delivery (SVD) or CS in their first delivery.

**Methods:**

This retrospective cohort study included women who underwent singleton deliveries at the International Peace Maternity and Child Health Hospital from January 2013 to December 2016. Data on the perinatal outcomes of all the women were extracted from the medical records. Multivariate logistic regression was conducted to assessed the association between CS in the first delivery and adverse perinatal outcomes in the subsequent pregnancy.

**Results:**

CS delivery in the subsequent pregnancy was more likely for women who underwent CS in their first birth than for women with previous SVD (97.3% versus 13.2%). CS in the first birth was also associated with a significantly increased risk of adverse outcomes in the subsequent pregnancy, especially in women who underwent a non-indicated CS. Adverse perinatal outcomes included pregnancy-induced hypertension [adjusted odds ratio (OR), 95% confidence interval (CI): 2.20, 1.59–3.05], gestational diabetes mellitus (1.82, 1.57–2.11), gestational anaemia (1.27, 1.05–1.55), placenta previa (3.18, 2.15–4.71), placenta accreta (2.75, 1.75–4.31), and polyhydramnios (2.60, 1.57–4.31) in the mother and preterm delivery (1.37, 1.06–1.78), low birth weight (3.78, 2.07–6.90), macrosomia (5.04, 3.95–6.44), and neonatal jaundice (1.72, 1.39–2.14) in the baby.

**Conclusions:**

CS in the first delivery markedly increases the risk of repeated CS and maternal-fetal complications in the subsequent pregnancy, especially in women with a non-indicated CS.

## Background

The high prevalence of caesarean section (CS) is a global public health issue. According to a WHO statement in 1985, regional CS rates should not exceed 10–15% [1, 2]. However, the rate of CS has markedly increased from approximately 6 to 40% in low, medium and high income countries in the past three decades [3–6]. China has a high rate of CS as well as CS without medical indication [4, 6]. Between 1988 and 2010, caesarean rates in all of China rose from 3.4 to 52.5%, while urban rates increased from 10 to 57% [7, 8]. Maternal request and previous CS are the most common indications for CS in mainland China [9]. According to a large, cross-sectional study of 496,054 caesarean deliveries, the prevalence of primary non-indicated CS increased from 0.6% in 1993–1995 to 21.3% in 2006–2010 [10]. Other studies have reported national rates of non-indicated CS ranging from 28.43 to 38.1% in China [9, 11]. Prior CS is an important factor in the determination of a subsequent CS, which has led to overall high CS rates [6]. As implementation of China’s ‘two-child policy’ has triggered the next baby boom, many women with a previous CS might require repeated CS in their subsequent deliveries [9, 12]. Thus, the adverse outcomes in subsequent pregnancies for Chinese women with a previous CS must be further surveyed.

Although CS is accepted as a fairly safe procedure, and the overall rates of CS complications have decreased during the last decade [13], CS is still associated with an increased risk of maternal complications. According to recent data up to 2015, the WHO announced that CS rates higher than 10% were not associated with reductions in maternal and newborn mortality rates at the population level [2, 14, 15]. The WHO global survey indicated that caesarean delivery was positively associated with an increased risk of postpartum antibiotic use, maternal morbidity and mortality, and fetal and neonatal morbidity [3, 4]. Furthermore, prior CS was significantly associated with a deficit in subsequent fertility [16–19] and an increased risk of unexplained stillbirth in the subsequent pregnancy [20, 21]. Repeated CS was reported to increase maternal morbidity, including placenta accreta, reduced fetal growth, preterm delivery, pelvic pain, and adverse reproductive effects [22, 23]. However, few studies [24–27] of the Chinese population have explored the association between previous CS and adverse perinatal outcomes in a subsequent pregnancy, especially in women with a first non-indicated CS. Thus, we designed this study to compare the perinatal outcomes of subsequent pregnancies in women with a first spontaneous vaginal delivery to those with a CS.

## Methods

### Data source

The data for this study were obtained from the databases of the International Peace Maternity and Child Health Hospital. The perinatal database contains pregnancy, delivery, and pregnancy outcome data for all births submitted as paper copies of the delivery record or through an electronic extract in this hospital. Validation was performed on the data to check for errors and inconsistencies in documentation and coding. This study was performed in accordance with the declaration of Helsinki. Complications related to pregnancy and delivery, as well as related co-morbidities, were coded using criteria taken from the 9th and 10th revisions of the International Classification of Diseases (ICD-9 and -10).

### Study population

In this retrospective study, we included all parous women who underwent a singleton delivery at the International Peace Maternity and Child Health Hospital from January 2013 to December 2016. Subjects with missing information on the previous mode of delivery, with multiple pregnancies in the first birth, or with parity over twice were excluded. To reduce heterogeneity of the study population, women were respectively excluded due to their foreign nationality and first instrumental vaginal delivery. We ultimately included women with spontaneous vaginal delivery (SVD group) and those with CS (CS group) in the first delivery into the analysis.

### Study design and definitions

A retrospective cohort study design was used, and there were two groups according to the first mode of delivery in the cohorts. In consideration of the effects of indications for first CS on the next pregnancy, a subgroup analysis was performed between the previous SVD group and previous non-indicated CS group. Potential confounders were extracted, including birth place, nationality, insurance, marital status, age at second delivery, height, body mass index (BMI), education level, gravidity, and interpregnancy interval. Age was given in completed years at the time of delivery and was further subgrouped into < 25, 25–29, 30–34, 35–39, and ≥ 40 years. Height was classified into < 155, 155–164.9, 165–174.9, and ≥ 175 cm. BMI was calculated by dividing antenatal booking weight (kg) by height squared (m) and was then recoded into the following five categories: < 18.5, 18.5 to 24.9, 25 to 29.9, and ≥ 30 kg/m^2^. Educational level was given as the higher level of the couple, which was divided into five groups (≤9, 10–12, 13–15, and ≥ 16 years). Interpregnancy interval (< 1, 1–3, 3–5, 5–10, and ≥ 10 years) was defined as the time elapsed from the date of the first delivery to the date of subsequent conception adjusted by ultrasound with the unit of measurement recorded in years. In terms of outcomes at first birth, women with pregnancy-induced hypertension (PIH), preeclampsia (PE), gestational diabetes mellitus (GDM), preterm delivery, low birth weight (LBW), macrosomia, or stillbirths in the first birth were compared in groups to assess differences.

In the second pregnancy, the following outcomes were assessed: PIH (diastolic blood pressure > 90 mmHg on two occasions 4 h apart or a single reading of > 110 mmHg from 20 weeks gestation), PE (PIH and proteinuria 0.3 g/24 h) [28, 29], GDM (diagnosed using a 2-h, 75 g oral glucose tolerance test) [30], gestational anaemia (anaemia occurred in subsequent pregnancy, excluding haematological diseases and history of anaemia), gestational thyroid dysfunction (thyroid dysfunction existed during the subsequent pregnancy, excluding surgery history of the thyroid, prior diagnosis of thyroid diseases, and taking medicines that influence thyroid function), intrahepatic cholestasis of pregnancy (ICP), premature rupture of membranes (PROM, defined as rupture of fetal membranes at least 1 h prior to the onset of labour) [31], fetal distress, meconium-staining of the amniotic fluid, amniotic fluid volume (AFV), placental abruption and placenta previa (placental abruption was defined as premature separation of a normally implanted placenta from the uterus, while placenta previa was defined as implantation of the placenta over or near the internal opening of the cervix), placenta accreta (placenta accreta refers to the entire spectrum of conditions, including accreta, increta, and percreta, as well as to cases of clinically apparent morbidly adherent placenta) [32, 33], rupture of the uterus, antepartum haemorrhage (APH), fetal growth restriction (FGR), abnormal fetal position, dystocia, antepartum fever, mode of delivery (spontaneous vaginal, instrumental, or CS) in subsequent pregnancy, postpartum haemorrhage (PPH; blood loss > 500 ml for vaginal delivery, > 1000 ml for caesarean), and puerperal infection. Neonatal outcomes included stillbirth (delivery of a dead baby at or after 24^+ 0^ weeks gestation), preterm (24^+ 0^ to 36^+ 6^ weeks of gestation), LBW (< 2500 g), macrosomia (> 4000 g), Apgar score at 1 min, neonatal infection, neonatal death (death of a liveborn infant in the first 4 weeks of life), admission to a neonatal intensive care unit, neonatal respiratory distress syndrome (NRDS), transient tachypnea of newborn (TTN), neonatal jaundice, and birth injury.

The primary indications for CS were divided into 3 categories: maternal indications, fetal indications, and maternal request with no obstetric reasons. To clarify these indications, we divided caesarean deliveries into 2 categories, medically indicated or non-medically indicated [11]. Medically indicated CS included fetal distress; prolonged labour (dystocia); cephalopelvic disproportion; malpresentation; PE or eclampsia; haemolysis, elevated liver enzyme, and low platelets syndrome (HELLP); ICP; placental abruption; placenta praevia; scarred uterus; oligohydramnios; intrauterine infection; and suspected macrosomia. Non-medically indicated CS was defined as a primary CS done on maternal request without medical indication (CDMR) or a physician documented “social influence”. These included: elderly primigravida as the only indication, a “precious” infant-defined as in vitro pregnancy or poor obstetric history, severe myopia, isolated PROM without fetal heart rate (FHR) abnormalities, request for concomitant myomectomy or ovarian cystectomy, or other factors (isolated chronic hypertension; gestational hypertension; diabetes mellitus without macrosomia).

### Statistical analyses

Statistical analyses were performed using SPSS version 20 software (IBM, SPSS Statistics, Armonk, NY, USA). Descriptive statistics were reported as the means and standard deviations for continuous variables and percentages for categorical variables. Differences in demographics and first birth outcomes in the cohorts were assessed using the Pearson Chi-square test or Fisher’s exact test. Odds ratios (ORs) with their 95% confidence intervals (CIs) were calculated to evaluate the association between CS in the first delivery and perinatal outcomes in the subsequent pregnancy via univariate (unadjusted for covariates) and multivariate statistical methods (adjusted for potential confounders to ascertain that relationships were due to demographic characteristics or adverse obstetric events). Variables found to be statistically significant in univariate analysis and confirmed as a risk factor by published literature were entered into the multivariate logistic regression model. Statistical significance was set at *P* < 0.05.

## Results

**Fig. 1:**
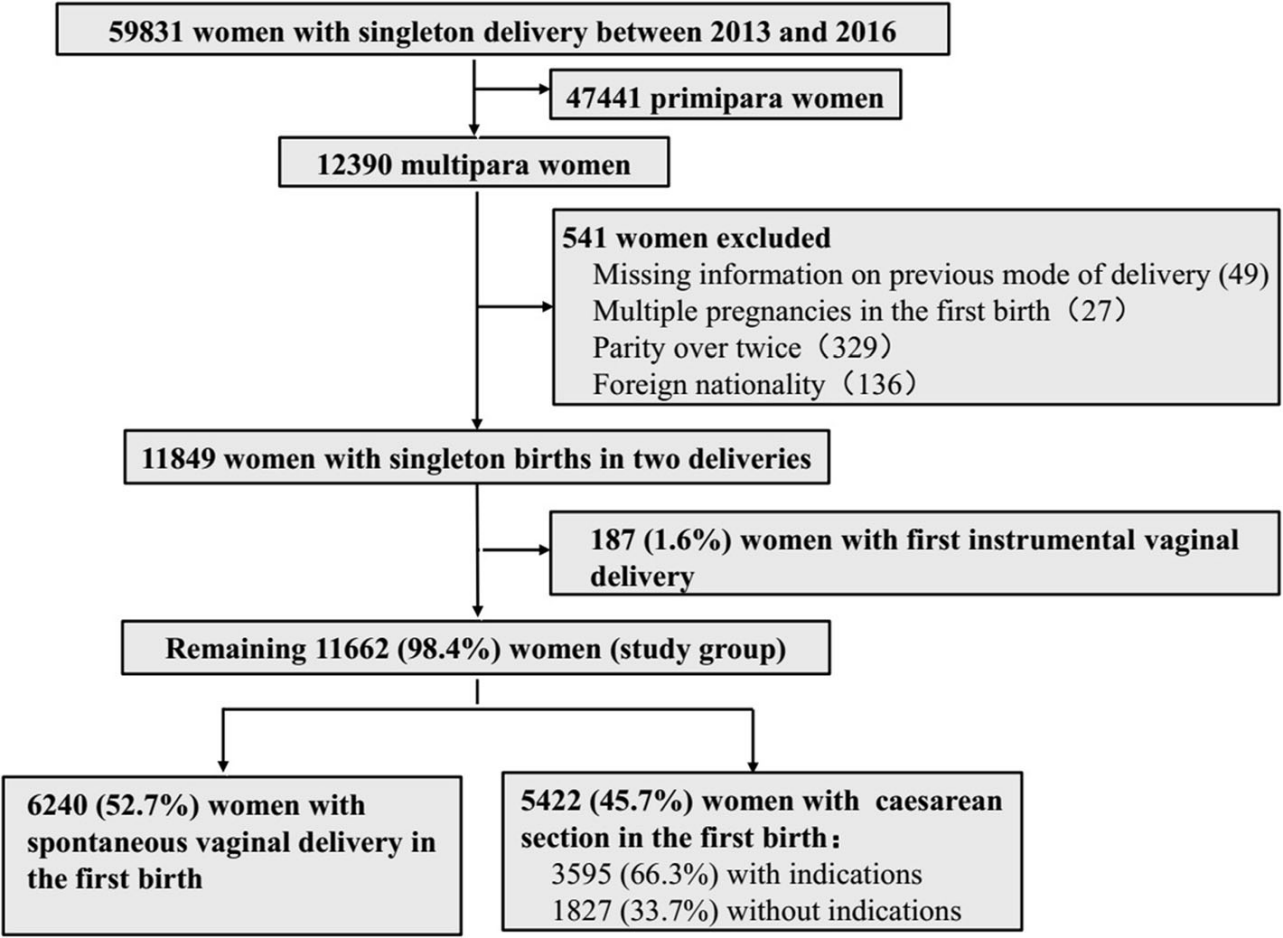
Flow chart showing study population in cohorts

In total, 59,831 singleton births were recorded in the International Peace Maternity and Child Health Hospital between January 2013 and December 2016, and 12,390 who had a delivery history were treated as potential subjects. After excluding 49 women with missing information on the previous mode of delivery, 21 women with multiple pregnancies in the first birth, 329 women with parity over twice, 136 women with foreign nationality, and 187 women with instrumental vaginal delivery in the first birth, 11,662 subjects were included into the final analysis (6240 in the SVD group and 5422 in the CS group). Among the 5422 women in the CS group, 3595 (66.3%) women had medical indications for CS, and 1827 (33.7%) women had no medical indications for CS in their first birth (Fig. 1).

The demographic characteristics among the groups are compared in Table 1. Significant differences were detected when comparing the CS group with the SVD group, including insurance, age at the subsequent delivery, height, maternal BMI, educational level, gravidity, and interpregnancy interval, while no differences in race were observed.

**Table 1:** Maternal baseline characteristics and the first birth characteristics among women with previous SVD and those with previous CS and non-indicated CS

	previous SVD (*n* = 6240)		previous CS (*n* = 5422)		*P-* value^a^	previous non-indicated CS (*n* = 1827)		*P-* value^b^
	N	%	N	%		N	%	
Baseline characteristics								
Birth place								
Shanghai	4753	76.2%	3991	73.6%	0.001	1413	77.3%	0.300
Out of Shanghai	1487	23.8%	1431	26.4%		414	22.7%	
Race								
Han	6164	98.8%	5353	98.7%	0.791	1812	99.2%	0.158
Minority	76	1.2%	69	1.3%		15	0.8%	
Insurance								
Public	4239	67.9%	3376	62.3%	< 0.001	1099	60.2%	< 0.001
Self-pay	2001	32.1%	2046	37.7%		728	39.8%	
Marital status								
Married/cohabitating	6158	99.7%	5366	99.5%	0.093	1802	99.3%	0.007
Not married/cohabitating	17	0.3%	25	0.5%		13	0.7%	
Age at the second delivery								
<25 years	73	1.2%	39	0.8%	< 0.001	10	0.5%	< 0.001
25–29 years	1152	18.5%	739	13.6%		228	12.5%	
30–34 years	3055	48.9%	2727	50.3%		917	50.2%	
35–39 years	1702	27.3%	1693	31.2%		585	32.0%	
≥ 40 years	258	4.1%	224	4.1%		87	4.8%	
Height (cm)								
<155	222	3.6%	317	5.8%	< 0.001	91	5.0%	0.001
155–164.9	4013	64.3%	3651	67.4%		1206	66.0%	
165–174.9	1935	31.0%	1419	26.2%		520	28.5%	
≥ 175	70	1.1%	35	0.6%		10	0.5%	
Maternal BMI (Kg/m2)								
<18.5	550	8.8%	405	7.5%	< 0.001	139	7.6%	< 0.001
18.5–24.9	5313	85.2%	4530	83.5%		1507	82.5%	
25–29.9	338	5.4%	423	7.8%		155	8.5%	
≥ 30	39	0.6%	64	1.2%		26	1.4%	
Educational level (years)								
≤ 9	266	4.4%	205	3.9%	0.042	35	2.0%	< 0.001
10–12	494	8.2%	469	9.0%		130	7.4%	
13–15	1205	20.1%	1119	21.6%		398	22.5%	
≥ 16	4049	67.3%	3399	65.5%		1203	68.1%	
Gravidity								
≤ 2	2984	47.8%	2366	43.6%	< 0.001	825	45.2%	0.045
≥ 3	3256	52.2%	3056	56.4%		1002	54.8%	
Interpregnancy interval (years)								
<1	431	7.0%	88	1.6%	< 0.001	29	1.6%	< 0.001
1–3	1935	31.2%	1350	25.0%		446	24.5%	
3–5	1357	21.9%	1521	28.2%		540	29.6%	
5–10	1816	29.3%	1957	36.2%		665	36.5%	
≥ 10	657	10.6%	485	9.0%		142	7.8%	
First birth characteristics								
PIH								
Yes	109	1.7%	103	1.9%	0.538	65	3.6%	< 0.001
No	6131	98.3%	5319	98.1%		1762	96.4%	
PE								
Yes	81	1.3%	90	1.7%	0.105	40	2.2%	0.006
No	6159	98.7%	5332	98.3%		1787	97.8%	
GDM								
Yes	415	6.7%	378	7.0%	0.492	159	8.7%	0.003
No	5825	93.3%	5044	93.0%		1668	91.3%	
Stillbirths								
Yes	61	1.0%	10	0.2%	< 0.001	0	0.0%	< 0.001
No	6179	99.0%	5412	99.8%		1827	100.0%	
LBW								
Yes	144	2.3%	144	2.7%	0.227	26	1.4%	0.021
No	6096	97.7%	5278	97.3%		1801	98.6%	
Macrosomia								
Yes	279	4.5%	572	10.5%	< 0.001	211	11.5%	< 0.001
No	5961	95.5%	4850	89.5%		1616	88.5%	
Preterm delivery								
Yes	266	4.3%	188	3.5%	0.027	16	0.9%	< 0.001
No	5974	95.7%	5234	96.5%		1811	99.1%	

*SVD* spontaneous vaginal delivery, *CS* caesarean section, *BMI* body mass index, *PIH* pregnancy-induced hypertension, *PE* preeclampsia, *GDM* gestational diabetes mellitus, *LBW* low birth weight

^a^Comparison between previous CS and previous SVD

^b^Comparison between previous non-indicated CS and previous SVD

A comparison of the characteristics of the first birth is presented in Table 1. Women with an initial caesarean delivery were significantly more likely to have macrosomia (first CS group vs. first SVD group: 10.5% vs. 4.5%; *P* < 0.001) in the first birth than those in the SVD group. In contrast, women with an initial CS were less likely to have preterm delivery (first CS group vs. first SVD group: 3.5% vs. 4.3%; *P* = 0.027) and stillbirths (first CS group vs. first SVD group: 0.2% vs 1.0%; P < 0.001) in the first birth than those in the SVD group. There were no differences between the two groups in PIH, PE, GDM, or LBW in the first birth. However, there were significant differences in all first birth outcomes when comparing previous non-indicated CS to previous SVD.

**Fig. 2:**
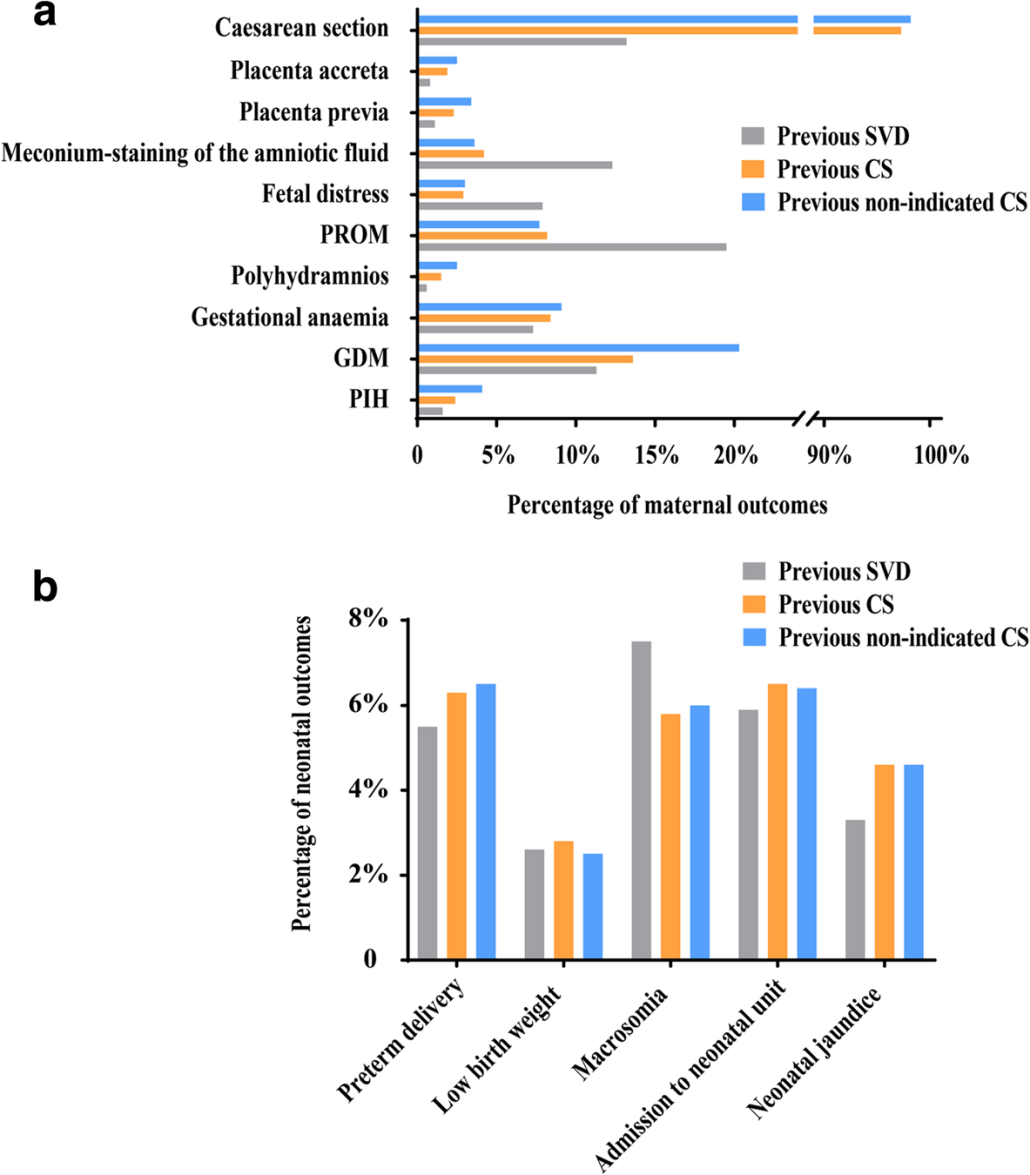
Incidences of the main perinatal outcomes of the second births. **a** Percentages of the main maternal outcomes of the second births among the three groups. **b** Percentages of the main neonatal outcomes of second births among the three groups. SVD: spontaneous vaginal delivery; CS: caesarean section; PIH: pregnancy-induced hypertension; PE: preeclampsia; GDM: gestational diabetes mellitus; PROM: premature rupture of membranes

Table 2 displays the associations between caesarean delivery in the first birth and maternal outcomes in the subsequent pregnancy, and the percentages of maternal outcomes are presented in Fig. 2a. After adjusting for potential confounders, women with previous CS were 1.38 times more likely to have PIH (95% CI: 1.04–1.82), 1.15 times more likely to have GDM (95% CI: 1.02–1.29), 1.72 times more likely to have ICP (95% CI: 1.04–2.84), 2.50 times more likely to have polyhydramnios (95% CI: 1.69–3.69), 2.11 times more likely to have placenta previa (95% CI: 1.52–2.94), 2.11 times more likely to have placenta accreta (95% CI: 1.47–3.04), 364.21 times more likely to choose caesarean delivery (95% CI: 294.74–450.06), and 8.3 times more likely to suffer instrumental vaginal delivery (95% CI: 4.41–15.6) in the next pregnancy. In contrast, the previous CS women were 66% less likely to have PROM (95% CI: 0.30–0.39), 67% less likely to suffer from fetal distress (95% CI: 0.27–0.40), 51 and 85% less likely to have moderate or severe meconium-staining of the amniotic fluid (95% CI: 0.39–0.62 and 0.11–0.19, respectively), respectively, and 46% less likely to have antepartum fever (95% CI: 0.33–0.89) in the next pregnancy. Given the risk of the first CS with medical indications in the subsequent pregnancy, we performed a subgroup analysis of pregnancy outcomes in women who did not have indications in the first delivery. In this subgroup, women with previous non-indication CS were more likely to experience PIH (adjusted OR: 2.20; 95% CI: 1.59–3.05), GDM (adjusted OR: 1.82; 95% CI: 1.57–2.11), gestational anaemia (adjusted OR: 1.27; 95% CI: 1.05–1.55), polyhydramnios (adjusted OR: 2.60; 95% CI: 1.57–4.31), placenta previa (adjusted OR: 3.18; 95% CI: 2.15–4.71), placenta accreta (adjusted OR: 2.75; 95% CI: 1.75–4.31), and CS (adjusted OR: 694.16; 95% CI: 468.45–1028.62). Seven women had uterine rupture in a subsequent pregnancy in the CS group, four of whom had a non-indicated CS in the first birth, but none of the women in the SVD group had uterine rupture.

**Table 2:** Association between previous caesarean delivery and maternal outcomes in the subsequent pregnancy

Maternal outcomes	previous SVD(*n* = 6240)		previous CS(*n* = 5422)		Crude OR1 (95% CI)	Model 1 Adjusted OR1 (95% CI)	previous nonindicated CS(*n* = 1827)		Crude OR2 (95% CI)	Model 2 Adjusted OR2 (95% CI)
	N	%	N	%			N	%		
PIH	99	1.6%	131	2.4%	1.54 (1.18–2.00)	1.38 (1.04–1.82)^a^	75	4.1%	2.66 (1.96–3.60)	2.20 (1.59–3.05)^d^
PE	84	1.3%	90	1.7%	1.24 (0.92–1.67)	1.22 (0.88–1.69)^a^	34	1.9%	1.39 (0.93–2.08)	1.24 (0.79–1.94)^d^
GDM	705	11.3%	736	13.6%	1.23 (1.10–1.38)	1.15 (1.02–1.29)^a^	371	20.3%	2.00 (1.74–2.30)	1.82 (1.57–2.11)^d^
Gestational anaemia	455	7.3%	458	8.4%	1.17 (1.03–1.34)	1.13 (0.98–1.30)	166	9.1%	1.27 (1.06–1.53)	1.27 (1.05–1.55)^d^
Gestational thyroid disease	256	4.1%	223	4.1%	1.01 (0.84–1.20)	1.03 (0.85–1.24)	64	3.5%	0.85 (0.64–1.12)	0.86 (0.65–1.16)^d^
ICP	29	0.5%	43	0.8%	1.71 (1.07–2.75)	1.72 (1.04–2.84)	14	0.8%	1.65 (0.87–3.14)	1.65 (0.84–3.23)^d^
PROM	1217	19.5%	445	8.2%	0.37 (0.33–0.41)	0.34 (0.30–0.39)	140	7.7%	0.34 (0.29–0.41)	0.32 (0.26–0.39)^d^
fetal distress	493	7.9%	159	2.9%	0.35 (0.29–0.42)	0.33 (0.27–0.40)	55	3.0%	0.36 (0.27–0.48)	0.34 (0.25–0.46)^d^
meconium-staining of the amniotic fluid										
I degree	58	0.9%	50	0.9%	0.91 (0.62–1.33)	0.94 (0.63–1.40)	17	0.9%	0.91 (0.53–1.57)	0.97 (0.56–1.70)^d^
II degree	236	3.8%	114	2.1%	0.51 (0.41–0.64)	0.49 (0.39–0.62)	33	1.8%	0.44 (0.30–0.63)	0.39 (0.26–0.57)^d^
III degree	472	7.6%	67	1.2%	0.15 (0.12–0.19)	0.15 (0.11–0.19)	17	0.9%	0.11 (0.07–0.18)	0.11 (0.07–0.18)^d^
AFV										
Oligohydramnios	65	1.0%	67	1.2%	1.20 (0.85–1.69)	1.15 (0.81–1.64)	25	1.4%	1.33 (0.84–2.12)	1.37 (0.85–2.21)^d^
Polyhydramnios	39	0.6%	82	1.5%	2.45 (1.67–3.59)	2.50 (1.69–3.69)	27	1.5%	2.39 (1.46–3.92)	2.60 (1.57–4.31)^d^
Placental abruption	19	0.3%	7	0.1%	0.42 (0.18–1.01)	0.51 (0.21–1.25)	4	0.2%	0.72 (0.24–2.11)	0.92 (0.30–2.80)^d^
Placenta previa	66	1.1%	124	2.3%	2.19 (1.62–2.96)	2.11 (1.52–2.94)	62	3.4%	3.29 (2.31–4.67)	3.18 (2.15–4.71)^d^
Placenta accreta	53	0.8%	104	1.9%	2.28 (1.64–3.18)	2.11 (1.47–3.04)	45	2.5%	2.95 (1.97–4.40)	2.75 (1.75–4.31)^d^
Rupture of uterus	0	0.0%	7	0.1%	/	/	4	0.2%	/	/
APH	61	1.0%	59	1.1%	1.11 (0.78–1.60)	0.96 (0.64–1.43)	25	1.4%	1.41 (0.88–2.25)	1.13 (0.66–1.93)^d^
FGR	82	1.3%	70	1.3%	0.98 (0.71–1.35)	0.93 (0.65–1.33)	22	1.2%	0.92 (0.57–1.47)	0.89 (0.53–1.51)^d^
Abnormal fetal position	193	3.1%	194	3.6%	1.16 (0.95–1.42)	1.09 (0.88–1.35)	47	2.6%	0.83 (0.60–1.14)	0.77 (0.55–1.09)^d^
Dystocia	197	3.2%	196	3.6%	1.15 (0.94–1.41)	1.09 (0.88–1.34)	46	2.5%	0.79 (0.57–1.10)	0.73 (0.52–1.04)^d^
Antepartum fever	55	0.9%	27	0.5%	0.56 (0.36–0.89)	0.54 (0.33–0.89)	9	0.5%	0.56 (0.28–1.13)	0.60 (0.29–1.24)^d^
Mode of delivery										
Caesarean section	823	13.2%	5278	97.3%	263.28 (217.98–318.01)	364.21 (294.74–450.06)^b^	1795	98.2%	375.49 (261.30–539.58)	694.16 (468.45–1028.62)^e^
Instrumental delivery	80	1.3%	14	0.3%	7.18 (3.97–13.01)	8.30 (4.41–15.60)^b^	1	0.1%	2.15 (0.29–15.96)	6.40 (0.84–48.80)^e^
PPH	119	1.9%	107	2.0%	1.04 (0.80–1.35)	0.69 (0.42–1.14)^c^	43	2.4%	1.24 (0.87–1.76)	0.54 (0.30–0.97)^f^
Puerperal infection	16	0.3%	15	0.3%	1.08 (0.53–2.19)	0.99 (0.35–2.83)^c^	4	0.2%	0.85 (0.29–2.56)	0.50 (0.12–2.19)^f^

*SVD* spontaneous vaginal delivery, *CS* caesarean section, *PIH* pregnancy-induced hypertension, *PE* preeclampsia, *GDM* gestational diabetes mellitus, *ICP* intrahepatic cholestasis of pregnancy, *PROM* prelabour rupture of membranes, *AFV* amniotic fluid volume, *APH* antepartum haemorrhage, *PPH* postpartum haemorrhage, *FGR* fetal growth restriction, *OR* odds radio, *CI* confidence interval

Model 1: all ORs adjusted for birth place, insurance, age at the second delivery, height, maternal BMI, educational level, gravidity, interpregnancy interval

^a^ORs further adjusted for PIH, PE and GDM in first birth

^b^ORs further adjusted for PIH, GDM, ICP, gestational anaemia, PROM and meconium-staining of the amniotic fluid, AFV, fetal distress, placental abruption, placenta previa, placenta accreta, rupture of uterus and antepartum fever

^c^ORs further adjusted for PIH, GDM, ICP, gestational anaemia, PROM and meconium-staining of the amniotic fluid, AFV, fetal distress, placental abruption, placenta previa, placenta accreta, rupture of uterus, antepartum fever and mode of delivery

Model 2: all ORs adjusted for insurance, marital status, age at the second delivery, height, maternal BMI, educational level, gravidity, interpregnancy interval

^d^ORs further adjusted for PIH, PE and GDM in first birth

^e^ORs further adjusted for PIH, GDM, gestational anaemia, PROM, meconium-staining of the amniotic fluid, AFV, fetal distress, placenta previa, placenta accreta, rupture of uterus

^f^ORs further adjusted for PIH, GDM, gestational anaemia, PROM and meconium-staining of the amniotic fluid, AFV, fetal distress, placenta previa, placenta accreta, rupture of uterus and mode of delivery

Table 3 shows the association between previous caesarean delivery and neonatal outcomes in the subsequent pregnancy, and the percentages of neonatal outcomes are displayed in Fig. 2b. After adjusting for potential confounders, previous CS women were 1.37 times more likely to have preterm delivery (95% CI: 1.14–1.66), 4.25 times more likely to have a baby with LBW (95% CI: 2.73–6.62), 4.79 times more likely to have macrosomia (95% CI: 3.94–5.83), and 1.39 times more likely to have their baby admitted to a neonatal unit (95% CI: 1.16–1.65) compared with women who had a first SVD. Babies with TTN (adjusted OR: 1.93; 95% CI: 1.33–2.81) were more common in women with a first CS, as were neonatal jaundice (adjusted OR: 1.72; 95% CI: 1.39–2.14). However, there were no differences in stillbirth or neonatal death in the subsequent pregnancy between the previous CS and SVD groups (95% CI: 0.27–2.18 and 0.34–13.74, respectively). Given the risk of the first CS with medical indications in the subsequent pregnancy, we performed a subgroup analysis of neonatal outcomes in women who did not have indications in the first delivery. In this subgroup, women with previous non-indication CS were 1.37 times more likely to have preterm delivery (95% CI: 1.06–1.78), 3.78 times more likely to have a baby with LBW (95% CI: 2.07–6.90), 5.04 times more likely to have macrosomia (95% CI: 3.95–6.44), and 1.58 times more likely to have a baby with neonatal jaundice (95% CI: 1.17–2.13) than women in the SVD group.

**Table 3:** Association between previous caesarean delivery and neonatal outcomes in the subsequent pregnancy

Neonatal outcomes	previous SVD(*n* = 6240)		previous CS(*n* = 5422)		Crude OR1 (95% CI)	Model 1 Adjusted OR1 (95% CI)	previous nonindicated CS(*n* = 1827)		Crude OR2 (95% CI)	Model 2 Adjusted OR2 (95% CI)
	N	%	N	%			N	%		
Stillbirths	13	0.2%	11	0.2%	0.97 (0.44–2.18)	0.76 (0.27–2.18)^a^	4	0.2%	1.05 (0.34–3.23)	0.68 (0.13–3.55)^a^
Preterm delivery	341	5.5%	343	6.3%	1.17 (1.01–1.36)	1.37 (1.14–1.66)^b^	119	6.5%	1.21 (0.97–1.50)	1.37 (1.06–1.78)^b^
LBW	162	2.6%	151	2.8%	1.08 (0.86–1.35)	4.25 (2.73–6.62)^c^	46	2.5%	0.97 (0.70–1.35)	3.78 (2.07–6.90)^c^
Macrosomia	466	7.5%	317	5.8%	0.77 (0.66–0.89)	4.79 (3.94–5.83)^d^	110	6.0%	0.79 (0.64–0.98)	5.04 (3.95–6.44)^d^
Apgar at 1 min<8	79	1.3%	74	1.4%	1.08 (0.78–1.49)	1.13 (0.77–1.66)	25	1.4%	1.08 (0.69–1.70)	1.18 (0.69–2.01)
Neonatal infection	83	1.3%	46	0.8%	0.64 (0.44–0.91)	0.81 (0.54–1.23)	12	0.7%	0.49 (0.27–0.90)	0.51 (0.26–1.03)
Neonatal death	4	0.1%	4	0.1%	1.15 (0.29–4.60)	2.17 (0.34–13.74)	1	0.1%	0.85 (0.10–7.64)	0.70 (0.02–34.51)
Admission to neonatal unit	366	5.9%	355	6.5%	1.12 (0.97–1.31)	1.39 (1.16–1.65)	117	6.4%	1.10 (0.89–1.36)	1.20 (0.94–1.54)
NRDS	13	0.2%	21	0.4%	1.86 (0.93–3.72)	1.88 (0.83–4.24)	7	0.4%	1.84 (0.73–4.62)	1.49 (0.48–4.61)
TTN	71	1.1%	91	1.7%	1.48 (1.09–2.03)	1.93 (1.33–2.81)	28	1.5%	1.35 (0.87–2.10)	1.55 (0.93–2.58)
Neonatal jaundice	206	3.3%	249	4.6%	1.41 (1.17–1.70)	1.72 (1.39–2.14)	84	4.6%	1.41 (1.09–1.83)	1.58 (1.17–2.13)
Birth injury	16	0.3%	9	0.2%	0.65 (0.29–1.49)	0.85 (0.35–2.06)	4	0.2%	0.85 (0.29–2.56)	0.85 (0.25–2.85)

*LBW* low birth weight, *NRDS* neonatal respiratory distress syndrome, *TTN* transient tachypnea of newborn(TTN)

Model 1: all ORs adjusted for birth place, insurance, age at the second delivery, height, maternal BMI, educational level, gravidity, interpregnancy interval, PIH, GDM, ICP, gestational anaemia, PROM and meconium-staining of the amniotic fluid, AFV, fetal distress, placental abruption, placenta previa, placenta accreta, rupture of uterus, antepartum fever

Model 2: all ORs adjusted for insurance, marital status, age at the second delivery, height, maternal BMI, educational level, gravidity, interpregnancy interval, PIH, GDM, gestational anaemia, PROM and meconium-staining of the amniotic fluid, AFV, fetal distress, placenta previa, placenta accreta, rupture of uterus

^a^ORs further adjusted for stillbirth at the first birth

^b^ORs further adjusted for preterm delivery at the first birth

^c^ORs further adjusted for LBW at the first birth

^d^ORs further adjusted for macrosomia at the first birth

## Discussion

### Main findings

Our results indicate that having a CS in the first singleton pregnancy is associated with an increased risk of adverse maternal and neonatal outcomes in subsequent singleton pregnancy in comparison to an initial SVD. The outcomes included a higher risk of PIH, GDM, ICP, polyhydramnios, placental previa, placental accreta, repeated caesarean delivery in the mother and preterm delivery, LBW, macrosomia, admission to a neonatal unit, TTN, and jaundice in the baby. Notably, in women without indications for CS in the first birth, there was still a significantly increased risk of PIH, GDM, polyhydramnios, placental previa, placental accrete, repeated caesarean delivery, and preterm, LBW, macrosomia, and neonatal jaundice.

### Strengths and limitations

To the best of our knowledge, this is one of the few epidemiological studies exploring the association between CS in the first birth and perinatal complications in the subsequent pregnancy. There are several advantages to our study. First, the data analysed were taken from a geographically stable population and collected at the time of delivery from case notes, thereby minimizing selection and recall bias. Second, changes in clinical practice are unlikely to influence the findings, as the data of the two groups were recorded during the same period. Last but not least, we restricted our analyses to women with a second singleton birth, which eliminated potential confounding effects of parity and multiple gestations, and we also performed a subgroup analysis between previous non-indicated CS and previous SVD to avoid the risk of CS indications in the next pregnancy. Still, we should note that the presence of missing values and the retrospective nature of data collection may have biased the results to some extent. Briefly, the lack of adverse maternal outcomes in the first birth, such as PROM, placenta previa, and APH, which may be risk factors that need to be adjusted to adverse outcomes in the next pregnancy, may have influenced the results. It was difficult to confirm the indications for CS from the retrospective data. Further prospective studies are required to reduce the information bias. Additionally, as the study was restricted to a single centre in Shanghai, the results may only be generalizable to that local area in China.

### Interpretation

In our study population, 45.7% of the subjects experienced CS in the first singleton birth, and 33.7% of them were non-indicated. The main reason for the high rate of CS and non-indicated CS may be attributed to the One Child Policy of the Chinese government during the period from 1978 to 2015. In the second singleton birth, the CS rate reached 52.3%, which was approximately three times the proportion recommended by the WHO. This finding is consistent with studies reported previously in China [9, 34]. We confirmed that some of the known baseline characteristics were associated with delivery mode, including maternal age, height, maternal BMI, educational level of the couples, gravidity, and interpregnancy interval, but these factors are not specific and were associated with a few of the adverse obstetric outcomes. Our present study findings suggest that CS in the first birth is linked with adverse maternal and neonatal outcomes in the next pregnancy. The effects of previous CS on the risk of placenta previa and placenta accreta are consistent with previous studies [24, 26, 27, 35–39]. These papers confirmed the association between placenta previa and previous CS and found that the ORs for placenta previa with one or more previous caesarean deliveries were 1.47–1.66, but the pooled OR of 2.2 from a meta-analysis [37] is consistent with the OR of 2.11 in our findings. Additionally, the risk of placenta accreta in our study was similar to results from previous research [27, 38]. Possible mechanisms for placenta previa and accreta following previous CS suggested by previous researchers have included failure of placental apparent migration, impaired differential growth of the lower uterine segment, and deficient decidua basalis in the presence of previous surgery injury [24, 40–42]. In the present study, we focused on the adverse outcomes in second births, and our study represents a homogeneous sample affected by previous CS, which ruled out the influence of parity. Although there is disagreement on the association of previous CS with a higher risk of placenta abruption in the second pregnancy in prior studies [24, 26, 27, 36, 38, 39], our study shows that previous CS does not increase the risk of placental abruption. Additionally, the incidence of uterine rupture (0.1%) in the previous CS cohort in our study was less than that reported in prior studies [26, 27, 43–46], which may be attributed to the lower rate of trial of labour in women with a first CS in our country. The risks of APH and PPH in our study were not associated with the initial caesarean delivery, which is not consistent with previous studies [26, 47]. Even in the subgroup analysis, there were no statistically significant differences for APH. However, the ORs were greater than one in the univariate analysis. There are several possible factors contributing to this finding: first, the missing information in the first birth may be confounding factors, and we cannot be certain whether they caused outcomes in the second birth; second, placental abruption, which is a primary cause of APH, was not increased in the first CS women in our study; third, the repeated CS reduced the occurrence of PPH.

Our study seems to be the first to document the associations between previous CS history and increased risk of PIH, GDM, ICP, and polyhydramnios in the next pregnancy. Especially in women without indications for a previous CS, the risks were much higher for PIH, GDM, and polyhydramnios. Similar research has shown the association between the first CS and the increased risk of PE [27, 48, 49], but no differences were found in our study. Prior CS has been shown to be an important risk factor for subsequent complications, including placental vasculopathies [50], which may lead to a high risk of PIH. Pregnant women with PIH and/or GDM in the first pregnancy are highly susceptible to recurrence in the next pregnancy [51, 52]. Thus, in our study, the high proportion of PIH and GDM in the first birth in the CS group may be a reason for the increased risk of PIH and GDM in the next pregnancy. Interestingly, when we regarded PIH and GDM in the first birth as confounding factors and adjusted for them, a high risk of PIH and GDM still existed in the next pregnancy for women with a first CS, especially in women without indications for the first CS. Moreover, uterine changes induced by prior CS may interfere with normal trophoblastic invasion and uteroplacental blood flow in subsequent pregnancies, resulting in abnormal placental function and subsequent gestational complications, including PIH and GDM [53, 54].

Compared to women with a prior SVD, the risks of PROM, fetal distress, meconium-staining of the amniotic fluid, and antepartum fever in the next birth were lower in women with an initial CS, which may be due to a missing confounding factor (PROM) in the first birth. Women with PROM were more likely to choose vaginal delivery in the first birth. Studies in the UK [31] and USA [55] showed that a PROM history was associated with a 6.6- to 20.6-fold increase in the risk of recurrent PROM. Furthermore, the decreased risk of fetal distress, meconium-staining of the amniotic fluid, and antepartum fever in women with primary CS in the second birth may also be related to the risks of these factors in the first birth.

The stillbirth rates were 2.0/1000 in both the previous CS group and previous SVD group, which was in accordance with preceding reports [26, 56], but there were no differences between the two groups in our research. Our findings on the effects of the first CS on the risk of preterm, LBW, macrosomia, admission to a neonatal unit, TTN of babies, and neonatal jaundice in the second pregnancy are consistent with previous studies [26, 43, 57, 58]. There are few published studies providing evidence to support our findings; therefore, it is difficult to interpret the risk of caesarean delivery on these outcomes. Although there was a higher risk of admission to a neonatal unit for babies of first CS women in the next pregnancy, it did not increase their neonatal death rate. Additionally, in the subgroup analysis, there was no difference in admission to a neonatal unit and TTN in the two groups.

With implementation of the “two-child policy” in China, an increasing number of families will choose to raise two children. To control the overall CS rate, it is important to decrease the occurrence of primary CS, especially elective CS with no medical indications. These study findings will help women and clinical doctors in making choices and balancing the risks and benefits of a caesarean delivery in the first and subsequent births. The decision for an elective primary caesarean delivery should be carefully considered for its impact on future births.

## Conclusions

CS in the first birth is associated with an increased risk of repeated caesarean delivery and adverse obstetric and perinatal outcomes in the following delivery, especially in women without CS indications. Clinicians might consider this information valuable when counselling women during pregnancy.

## Abbreviations

AFV: Amniotic fluid volume
APH: Antepartum haemorrhage
BMI: Body mass index
CI: Confidence interval
CS: Caesarean section
FGR: Fetal growth restriction
GDM: Gestational diabetes mellitus
HELLP: Haemolysis, elevated liver enzymes, and low platelets syndrome
ICP: Intrahepatic cholestasis of pregnancy
LBW: Low birth weight
NRDS: Neonatal respiratory distress syndrome
OR: Odds radio
PE: Preeclampsia
PIH: Pregnancy-induced hypertension
PPH: Postpartum haemorrhage
PROM: Premature rupture of membranes
SVD: Spontaneous vaginal delivery
TTN: Transient tachypnea of newborn
